# Proposal for an efficient, minimal electrodiagnostic protocol for confirmation of clinically diagnosed ulnar nerve entrapment neuropathy at the elbow

**DOI:** 10.3389/fneur.2025.1664216

**Published:** 2025-10-08

**Authors:** Anne Kurver, Jan Meulstee, Ronald H. M. A. Bartels, Wim I. M. Verhagen

**Affiliations:** ^1^Department of Neurology, Canisius Wilhelmina Hospital, Nijmegen, Netherlands; ^2^Department of Clinical Neurophysiology, Canisius Wilhelmina Hospital, Nijmegen, Netherlands; ^3^Department of Neurosurgery, RadboudUMC, Nijmegen, Netherlands

**Keywords:** UNE, ulnar nerve, electrodiagnostics, nerve conduction studies, protocol

## Abstract

**Introduction:**

The clinical diagnosis of ulnar nerve entrapment at the elbow can reliably be made based on typical clinical symptoms of UNE. In the present study, we constructed an efficient, minimal electrodiagnostic protocol for confirmation of clinically diagnosed UNE.

**Methods:**

A prospective cross-sectional cohort observational study was conducted among patients with clinical suspicion of UNE. In all, 210 arms were included, each examined according to a standard neurophysiological protocol.

**Results:**

Nerve conduction studies (NCS) indicated abnormalities in 60.5% of the cases. Of these, 84.3% had abnormal NCS results for the abductor digiti quinti muscle (ADV). The first dorsal interosseous muscle (FDI) and sensory NCS were abnormal in 68.5 and 59.1% of these cases, respectively.

**Conclusion:**

We recommend starting with NCS of the ADV for cases requiring only one abnormal test is needed to confirm the clinical diagnosis of UNE, followed by sensory NCS if the NCS of the ADV is normal.

## Introduction

Ulnar nerve entrapment at the elbow (UNE) is the second most prevalent entrapment neuropathy (1, 2). The reported incidence of UNE ranges from 8.9 to 25.2 per 100,000 person-years (3, 4). The clinical diagnosis can be reliably established based on typical clinical symptoms of UNE: pain and numbness in the ulnar half of the hand, along with weakness and atrophy of the ulnar innervated muscles (5). In the Netherlands, however, most surgeons prefer electrodiagnostic confirmation prior to surgery (6). Patients would benefit from a diagnostic protocol that minimizes discomfort. In the present study, therefore, we analyzed patient data and constructed an efficient, minimal electrodiagnostic protocol for confirmation of clinically diagnosed UNE, based on a prospective electrodiagnostic study in patients with clinically defined UNE.

## Methods

### Study design

We conducted a cross-sectional study among new patients (>18 years) who had been referred to our department for electrodiagnostic confirmation of the clinical diagnosis of UNE. We have used this database in previous studies (7). The only exclusion criteria was if the patient did not give consent to do all the NCS tests.

Criteria for the clinical diagnosis of UNE were numbness in the ulnar half of the fourth and fifth fingers, numbness in the ulnar half of the hand (palmar and dorsal) and/or weakness of the muscles in the hand innervated by the ulnar nerve (5). Patients were analyzed according to standard clinical procedures. For those with a bilateral clinical diagnose of UNE, both arms were included. The electrodiagnostic protocol was used to confirm the diagnosis.

We further analyzed the contribution of each electrodiagnostic criterion to the diagnosis of UNE.

### Ethical approval

The regional medical ethical commission (CMO Nijmegen – Arnhem) and the hospital ethical commission (LTC) approved this research project. Anonymised data were collected from patients receiving standard care. Given that no experimental procedures were applied, both ethical commissions deemed that no informed consent was necessary.

### Neurophysiological protocol

We followed the criteria of the American Association of Electrodiagnostic Medicine (AAEM) for UNE (8). A detailed description of the neurophysiological protocol for UNE has been published previously (7).

Motor and sensory NCS of the ulnar nerve were performed. At four sites the ulnar nerve was stimulated: wrist (W), below the elbow (BE), above the elbow (AE) and at the bicipital sulcus. A tape-measure was used to measure conduction distances, with an accuracy of 5 mm (9). The conduction distance across the elbow was predetermined at 8 cm (9). Based on previous research, this method and this distance was used to get best balance between effects of measurement-error in short distances and ‘dilution’ of the slowing due to long distances (9). The elbow was kept flexed at 90 degrees during the examination (10). Stimulation was controlled to be supramaximal. Surface electrodes were used to record compound muscle action potentials (CMAP). Sensory nerve action potentials (SNAP) were recorded antidromically using ring electrodes positioned around the fifth finger, with an electrode distance of 4 cm, but less in smaller fingers (range 2–4 cm) CMAP and SNAP amplitudes were measured from negative to positive top in mV and μV, respectively. All latencies were measured from stimulus to onset deflection from baseline. Special care was taken to find the optimal positions of the “active” recording electrode above the hypothenar and first dorsal interosseal space (FDI) by shifting its position during stimulation, in order to achieve a maximal amplitude and an initial deflection that was as sharp as possible. During the whole procedure, CMAP configuration was observed in order to minimize or, if necessary, correct an altered position of the hand. The conduction velocity of all segments was computed. The arm was warmed by warming pads prior to all tests (11). Target temperature was 34 °C, with a lower limit of 30 °C. Skin temperature was monitored by an infrared thermometer device. Cooled regions were warmed again.

Based on our previous findings (7), we did not include our needle EMG data. As stated in this previous paper, we used this neurophysiological approach exclusively for the confirmation of clinical UNE in a high prior-odds diagnostic setting. EMG has limited value for diagnosis UNE if NCS are normal, but is necessary for evaluating the differential diagnosis, this is however outside the scope of this paper.

We categorized and analyzed the parameters presented in Table 1.

**Table 1:** Electrodiagnostic criteria for UNE.

Motor
Stimulation at the wrist: absent CMAP of the ADV and/or FDI
Over the elbow (AE-to-BE) segment motor-NCV 10 m/s slower compared to lower arm segment (BE-to-wrist [W])
Decrease in ADV and/or FDI CMAP negative peak amplitude from BE to AE more than 20%.

Sensory
Absent distal SNAP (wrist)
Over the elbow (AE-to-BE) segment sensory-NCV 10 m/s slower compared to lower arm segment (BE-to-wrist [W])

ADV, abductor digiti quinti muscle; FDI, first dorsal interosseous muscle; CMAP, compound muscle action potential; SNAP, sensory nerve action potentials. Adapted from Kurver et al. (6).

### Statistical analyses

All statistical analyses were performed using SPSS version 29 (*Statistical Package for Social Sciences, Chicago, IL, USA*). We used descriptive statistics to analyze the demographic characteristics and results of motor and sensible NCS. Furthermore, we have used binomial tests to compare the results of AAEM EDX confirmed ulnar neuropathy (AECU) group with the whole group.

## Results

The sample included a total of 210 arms: 96 (45.7%) right; 114 (54.3%) left (see Table 2). The study population consisted of 91 men (43.3%) and 119 women (56.7.3%). The mean age of the patients was 54.5 ± 14.5 years. The NCS results were abnormal for 127 (60.5%) of the arms analyzed.

**Table 2:** Demographic characteristics.

Demographic characteristics	*N* = 210
Male-to-female ratio	91:119 (43.3%:56.7%)
Age (years)	
Mean ± standard deviation	53.7 ± 14.5
Range	19–91
Affected side (R/L)	96/114 (45.7%/54.3%)
UNE diagnosis according to AAEM criteria	127 (60.5%)

**Figure 1:**
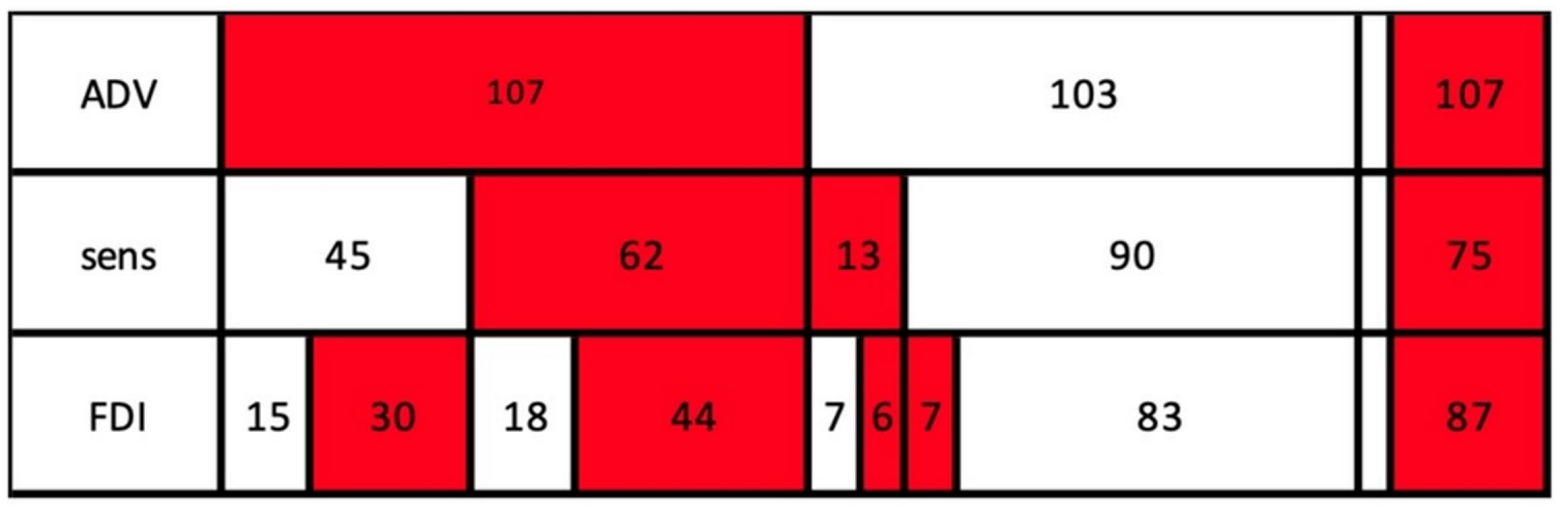
Distribution of NCS abnormalities. Red = abnormal NCS. White = normal NCS. ADV, abductor digiti quinti muscle NCS; FDI, first dorsal interosseous muscle NCS; sens, sensory NCS.

An overview of the NCS abnormalities found is presented in Table 3. Abnormalities in the ADV were present in 84.3% of the cases of AAEM EDX confirmed ulnar neuropathy (AECU), with FDI and sensory abnormalities found less frequently (68.5 and 59.1%, respectively). The distribution of NCS abnormalities is presented in Figure 1.

**Table 3:** Overview of NCS abnormalities.

NCS abnormalities	Frequency	% of total group (*N* = 210)	% of AECU group (*n* = 127)	*p*-value
ADV abnormalities	107	51.0%	84.3%	<0.001
FDI abnormalities	87	41.4%	68.5%	<0.001
Sensory abnormalities	75	35.7%	59.1%	<0.001
No NCS abnormalities	83	39.5%	0	n.a.

NCS, nerve conduction studies; ADV, abductor digiti quinti muscle; FDI, first dorsal interosseous; AECU, AAEM EDX confirmed ulnar neuropathy.

The contribution of each criterion within the group of patients with NCS abnormalities is displayed in Table 4. Slowing of the motor nerve conduction velocity (NCV) was the most frequent NCS abnormality (89.9%; see Table 4), followed by slowing of the sensory NCV (44.1%; see Table 4). The other criteria were abnormal in only a small number of cases.

**Table 4:** Contribution of each criterium.

Motor			
		Frequency	Percent
Absent wrist site CMAP of the:	ADV	3	2.4%
	FDI	3	2.4%
	ADV or FDI	4	3.1%
An AE-to-BE segment greater than 10 m/s slower than the BE-to-wrist (W) segment of the:	ADV	103	81.1%
	FDI	83	65.4%
	ADV or FDI	114	89.8%
Decrease in CMAP negative peak amplitude from BE to AE greater than 20% of the:	ADV	1	0.8%
	FDI	1	0.8%
	ADV or FDI	2	1.6%

Sensory		
	Frequency	Percent
Absent distal SNAP	9	7.1%
An AE-to-BE segment greater than 10 m/s slower than the BE-to-wrist (W) segment of the sensory nerve velocity	56	44.1%

ADV, abductor digiti quinti muscle; FDI, first dorsal interosseous muscle; CMAP, compound muscle action potential; SNAP, sensory nerve action potential.

In the AAEM guideline another criterium is mentioned for the diagnosis of UNE: “Absolute motor nerve conduction velocity (NCV) from above elbow (AE) to below elbow (BE) of less than 50 m/s” (7). We have analyzed this criterium for the ADV muscle and we found only three patients with an absolute NCV in this segment less than 50 m/s in the group without the UNE diagnosis. All these three patients had only a slightly decreased NCV of 47 m/s.

## Discussion

As reported in our previous studies, electromyographic testing for the presence of denervation in the absence of conduction abnormalities does not contribute to the electrodiagnostic confirmation of clinically diagnosed UNE (7). In the current study, our objective was to further optimize the electrodiagnostic protocol for confirmation of clinically diagnosed UNE.

All results of the current study are presented in Figure 1, and all possible combinations can be derived. For cases requiring only one abnormal test for confirmation, NCS of the ADV was most promising (107 patients). For cases in which NCS of the ADV is normal, NCS of FDI or sensory NCS can follow. Both FDI and sensory NCS were abnormal in 13 patients (when ADV was normal).

For cases requiring at least two abnormal nerve conduction tests, the combination of NCS of the ADV and the FDI yielded the most abnormal test results (74 of the 107 patients), in contrast to the combination of ADV and sensory tests, which yielded abnormal results for only 62 of the 107 patients in this group. The combination of NCS of the FDI and sensory test identified only 50 patients with abnormal tests. If three abnormal tests are required for diagnosis, only 44 patients would meet this criterium.

**Figure 2:**
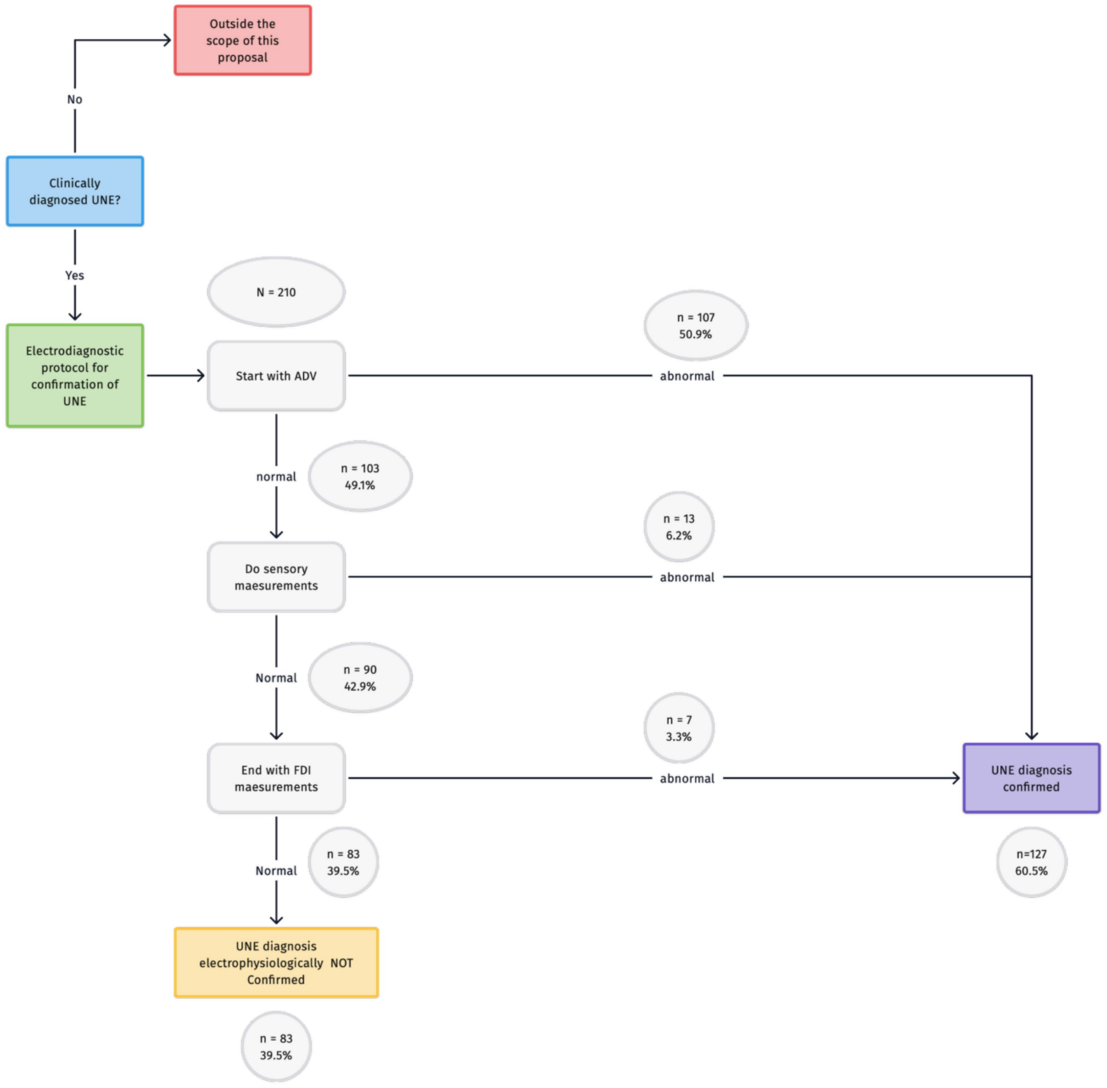
Proposal for an efficient electrodiagnostic protocol in the confirmation of UNE.

The results of our investigation are presented in Figure 2. Given that the most abnormalities were found in NCS of the ADV, this should be the starting point. If these results are normal, sensory NCS should follow. If the results of both of these tests are normal, the final step should be NCS of the FDI.

The choice of sensory NCS as the second step is not based solely on the numbers, given that sensory and FDI NCS are equally likely to yield abnormal results. In our experience, however, arriving at supramaximal stimulation in sensory NCS requires less stimulation strength than is the case for motor NCS. Patients might perceive this as less painful. However, the difference between pain perception in motor and sensory NCS is, to our knowledge, never investigated. Furthermore, given that the numbers are equal and the clinical symptoms are mainly sensory, we consider it appropriate to choose sensory NCS as a second step.

We must nevertheless note that, for cases requiring two abnormal tests, a combination of a motor and a sensory test is likely to be preferable to two motor tests, as the clinical symptoms are mainly sensory. Based on our data, however, the combination of two motor tests (ADV and FDI) yielded more abnormal results than did the combination of ADV and sensory NCS (74 vs. 62, respectively; see Figure 1).

In light of our preference for a minimal electrodiagnostic protocol for confirmation, one abnormal test should be sufficient; we propose this minimal electrodiagnostic protocol in a setting with well-defined clinically examined patients with the clinical diagnosis of UNE. If the clinical symptoms have a broad differential diagnosis (i.e., radiculopathy, plexopathy, polyneuropathy) a more extensive protocol including also electromyography is mandatory (12).

This study has several limitations that should be acknowledged. Firstly, we did not have specific exclusion criteria such as diabetes mellitus, prior surgery, or radiculopathy. The absence of these exclusions may have introduced confounding factors that could influence the electrodiagnostic findings and the generalisability of our results.

Secondly, the order of the diagnostic protocol was determined by the authors, with sensory NCS as the second step. However, this decision was not based on robust scientific evidence beyond the rationale provided above, which may limit the reproducibility of our approach in other settings.

Furthermore, it is noteworthy that 39.5% of patients in our cohort demonstrated normal findings on electrodiagnostic testing, despite a clinical diagnosis of ulnar neuropathy.

For such cases, we suggest using ultrasound of the ulnar nerve as the next step (12–14). If both of these tests are normal, another diagnosis should be considered. If the clinical symptoms are still consistent with ulnar neuropathy and possible further differential diagnosis have been excluded with more extensive neurophysiological studies including electromyography, the diagnosis can still be made, given the possibility of false negatives in both NCS and ultrasound. Within the context of CTS, it has been demonstrated that patients with negative NCS and ultrasound tests can still benefit from CTS treatment (15). The same could be true for patients with ulnar neuropathy, although further research is necessary to confirm this.

## Conclusion

We propose starting with NCS of the ADV for cases requiring only one abnormal test to confirm the clinical diagnosis of UNE. If these results are normal, sensory NCS should follow. For cases requiring at least two abnormal tests, the combination of NCS of the ADV and FDI would be most useful. If no abnormalities are found in NCS, an ultrasound of the ulnar nerve should be the next step. If both tests are normal, another diagnosis should be considered and more extensive electrophysiological studies including electromyography must be done. If the clinical symptoms are still consistent with ulnar neuropathy, however, normal test results should not completely rule out this diagnosis, given the possibility of false negative results in both of these tests.

## Data Availability

The raw data supporting the conclusions of this article will be made available by the authors without undue reservation.
